# *In Silico* Workflow for the Discovery of Natural Products Activating the G Protein-Coupled Bile Acid Receptor 1

**DOI:** 10.3389/fchem.2018.00242

**Published:** 2018-07-02

**Authors:** Benjamin Kirchweger, Jadel M. Kratz, Angela Ladurner, Ulrike Grienke, Thierry Langer, Verena M. Dirsch, Judith M. Rollinger

**Affiliations:** ^1^Department of Pharmacognosy, University of Vienna, Vienna, Austria; ^2^Department of Pharmaceutical Chemistry, University of Vienna, Vienna, Austria

**Keywords:** GPBAR1, TGR5, pharmacophore, virtual screening, natural product, triterpene

## Abstract

The G protein-coupled bile acid receptor (GPBAR1) has been recognized as a promising new target for the treatment of diverse diseases, including obesity, type 2 diabetes, fatty liver disease and atherosclerosis. The identification of novel and potent GPBAR1 agonists is highly relevant, as these diseases are on the rise and pharmacological unmet therapeutic needs are pervasive. Therefore, the aim of this study was to develop a proficient workflow for the *in silico* prediction of GPBAR1 activating compounds, primarily from natural sources. A protocol was set up, starting with a comprehensive collection of structural information of known ligands. This information was used to generate ligand-based pharmacophore models in LigandScout 4.08 Advanced. After theoretical validation, the two most promising models, namely BAMS22 and TTM8, were employed as queries for the virtual screening of natural product and synthetic small molecule databases. Virtual hits were progressed to shape matching experiments and physicochemical clustering. Out of 33 diverse virtual hits subjected to experimental testing using a reporter gene-based assay, two natural products, farnesiferol B (**27**) and microlobidene (**28**), were confirmed as GPBAR1 activators reaching more than 50% receptor activation at 20 μM with EC_50_s of 13.53 μM and 13.88 μM, respectively. This activity is comparable to that of the endogenous ligand lithocholic acid (**1**). Seven further virtual hits showed activity reaching at least 15% receptor activation either at 5 or 20 μM, including new scaffolds from natural and synthetic origin.

## Introduction

The G protein-coupled bile acid receptor 1 (GPBAR1), also commonly named M-BAR or Takeda G-protein-coupled receptor 5 (TGR5), is a rhodopsin-like G protein-coupled receptor (GPCR) expressed in various tissues. It is primarily present in the bile duct, digestive system, spleen, and placenta. It is a cell-surface receptor comprising an extracellular N-terminus, an intracellular C-terminus and seven trans-membrane helices connected by intra- and extracellular loops. Its endogenous ligands are bile acids and neurosteroids. The binding pocket is predicted to be located between the trans-membrane helices. Next to the transcription factor farnesoid X receptor (FXR), GPBAR1 was the second receptor discovered to be responsive to bile acids (Maruyama et al., 2002; Kawamata et al., 2003; Keitel et al., 2010; Gertzen et al., 2015).

In the past decade, this receptor has attracted attention as a potential drug target for a variety of pathologic conditions (Hodge and Nunez, 2016), predominantly because GPBAR1 is a key receptor in the adjustment of energy expenditure and glucose metabolism with possible implications for the treatment of obesity and type 2 diabetes. Its activation in enteroendocrine L-cells leads to the release of the incretins peptide tyrosine tyrosine (PYY) and glucagon like peptide 1 (GLP1), which promote insulin secretion in the pancreas and are important in the suppression of appetite (Woods and D'Alessio, 2008; Bala et al., 2014). GPBAR1 activation in pancreatic cells leads to an enhanced insulin secretion and a recovery of β-cell mass and function switching from glucagon to GLP1 (Kumar et al., 2012, 2016). In striated myocytes and brown adipocytes, GPBAR1 activation leads to thyroid hormone activation. In white adipocytes it mediates remodeling into beige cells and improves mitochondrial dynamics and cellular respiration rate (Watanabe et al., 2006; Velazquez-Villegas et al., 2018). Moreover, endothelium- and liver-protecting, as well as immunosuppressing effects offer perspectives for new therapies for diseases like atherosclerosis and inflammatory liver diseases (Keitel et al., 2007, 2008; Keitel and Haussinger, 2011; Pols et al., 2011; Asgharpour et al., 2015). Unusual for GPCRs, the GPBAR1 seems to only transfer signaling via G proteins and not via β-arrestins (Jensen et al., 2013).

In animal trials, GPBAR1 agonists showed promising results, however, difficulties have also been encountered since GPBAR1 agonists may induce itching and gallbladder extension (Vassileva et al., 2006; Alemi et al., 2013). Interestingly, gallbladder extension upon GPBAR1 activation is mainly caused by smooth muscle relaxation via induction of the cAMP–PKA pathway independent of the agonist scaffold (Lavoie et al., 2010; Li et al., 2011). The plethora of GPBAR1-mediated biological functions appears to be an obvious opportunity, but a major drawback at the same time (Vassileva et al., 2006; Alemi et al., 2013; Hodge and Nunez, 2016). Novel agonistic scaffolds may incorporate a different and possibly more favorable side effect profile in terms of receptor and functional selectivity as well as pharmacokinetic properties. In this sense there is a demand for new GPBAR1 ligands as they may help to cope with pharmacologically unmet therapeutic needs against metabolic diseases.

**Figure 1:**
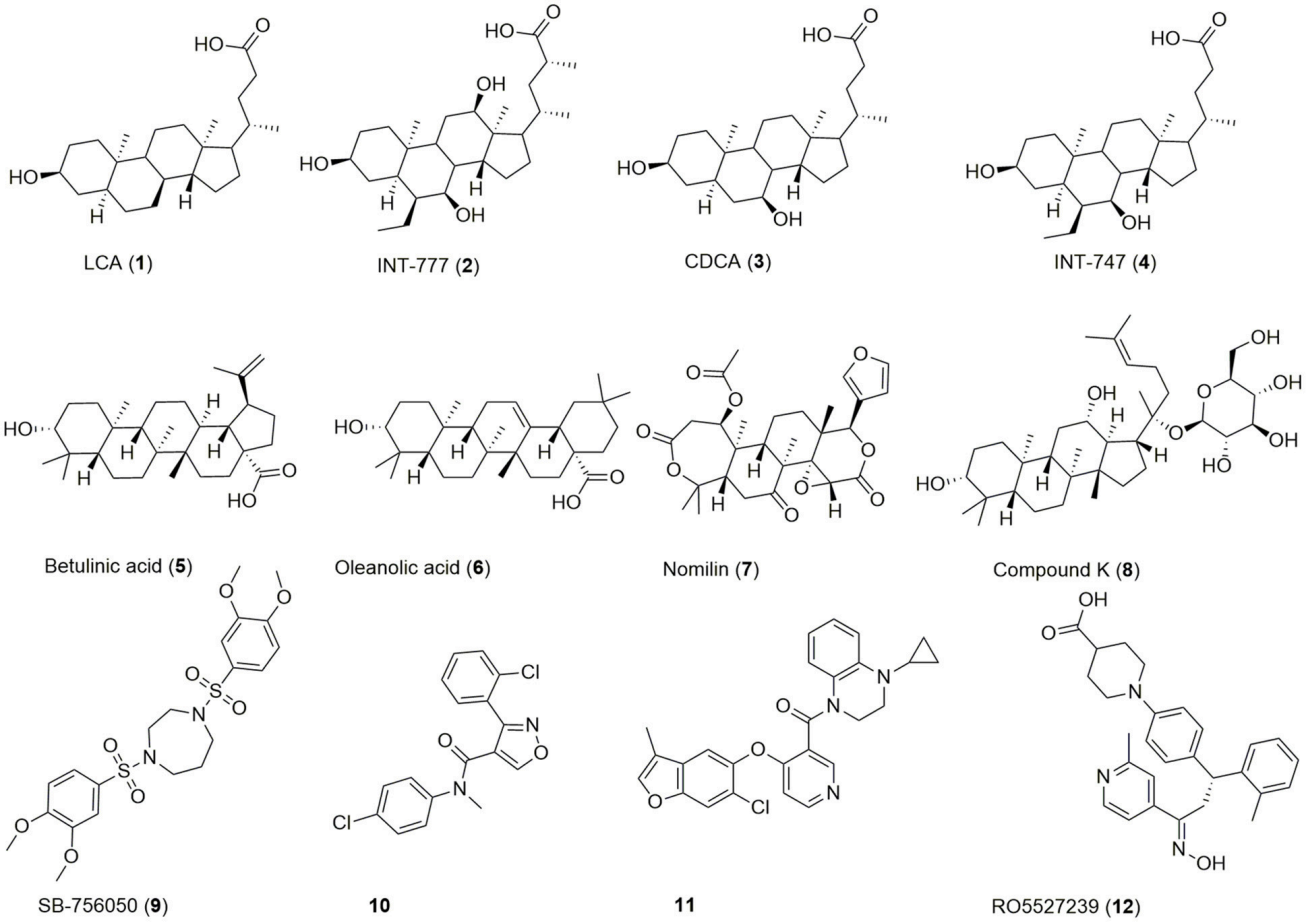
Chemical structures of examples for GPBAR1 agonists.

Beside bioassay-guided fractionation of plant extracts (Sato et al., 2007), bioisosteric replacement (Park et al., 2014), and exploitation/lead optimization of bile acid scaffolds (Pellicciari et al., 2009), previous efforts in the discovery of GPBAR1 modulators have focused on high throughput screening (HTS) (Evans et al., 2009; Herbert et al., 2010; Londregan et al., 2013; Martin et al., 2013) leading to a broad range of agonists of which some are depicted in Figure 1.

Chenodeoxycholic acid (CDCA, **3**) is a primary bile acid, which activates both GPBAR1 and the nuclear receptor FXR. By bacterial dehydroxylation, CDCA is transformed into the more potent lithocholic acid (LCA, **1**). The bile acid's potency on GPBAR1 can be further increased by conjugation with glycine or taurine, whereas taurine-conjugated lithocholic acid (TLC) is the most potent endogenous ligand (Sato et al., 2008). Lead optimization of the bile acid scaffold led to INT-747 (**4**), an approved drug for the treatment of primary biliary cholangitis and dual agonist of GPBAR1 and FXR as well as INT-777 (**2**), a more selective GPBAR1 agonist (Pellicciari et al., 2009; Fiorucci et al., 2014; Floreani and Mangini, 2018). Beside bile acids, several secondary plant metabolites activate this receptor (**5**-**8**). The antidiabetic effect of e.g., *Olea europaea* L. leaves may be linked to GPBAR1 activation by its major constituent oleanolic acid (**6**) (Sato et al., 2007). Moreover, HTS and extensive SAR efforts gave access to a large range of synthetic compounds activating this receptor in the nanomolar and low micromolar range (**9**-**12**).

Absence of a crystal structure of GPBAR1 forced researchers to rely either on homology models or ligand-based approaches for *in silico* studies, as this is the case for most GPCRs (Peeters et al., 2011; Vaidehi et al., 2014). Several GPBAR1 homology models have been described up to now. They all represent fundamentally different bile acid binding poses, but none of them is able to cover all results from mutagenesis studies (Macchiarulo et al., 2013; D'Amore et al., 2014; Gertzen et al., 2015; Yu et al., 2015). This prompted us to develop a ligand-based approach using pharmacophore modeling for the identification of new GPBAR1 agonists.

Pharmacophore modeling and subsequent virtual screening (VS) is a well-established method in the early drug discovery process showing some important benefits: (1) pharmacophore screening can retrieve ligands with structurally diverse scaffolds and allows for so called “scaffold-hopping”; (2) it can automatically and rapidly filter large compound libraries; (3) ligand-based pharmacophore VS has been able to retrieve satisfactory results, also without structural information on the target (Evers et al., 2005; Ha et al., 2015; Akram et al., 2017). Here, we report on the construction of two ligand-based 3D pharmacophore models, their *in silico* and *in vitro* validation, and the directed discovery of sesquiterpene coumarins as a new class of potent GPBAR1 agonists.

## Materials and methods

### Software

The generation of pharmacophore models, their subsequent refinement and VS was performed with LigandScout 4.08 Advanced, available by Inte:Ligand GmbH (Wolber and Langer, 2005). The conformational libraries for both pharmacophore modeling and the VS process were created with i:Con, LigandScout's implemented conformer generator (Friedrich et al., 2017). Shape comparison was performed with OpenEye's ROCS 3.2.1.4 (Hawkins et al., 2007; OpenEye, 2016). 2D structures were drawn with ChemDraw Professional 15.0.

### Data sources

For model generation in LigandScout, structural data of GPBAR1 ligands with bioactivity annotations were collected. The data for GPBAR1 available in the ChEMBL database was extracted on March 15th 2016. It consisted of 24 different publications with 623 reported EC_50_ values (Bento et al., 2014). The reliability of the content was checked with the original literature. This molecule set was extended by extracting data from another 18 publications, 24 patents and previous in house projects, resulting in a total of 1025 activity annotations.

### Decoy set

For the experimental validation of pharmacophores, next to a set of active molecules, also a set of inactive molecules and/or a set of decoys is necessary (Schuster et al., 2006). In contrast to true inactives, which are molecules reported in the literature not to be active at the target, decoys are hypothetical structures, which are unlikely to show activity at the target, but have not yet been tested experimentally. Due to the shortage of published negative data and therefore the presence of only a small set of reliably tested inactive compounds, a set of decoys was generated using the Dude decoys database (http://dude.docking.org/): 338 molecules from the “High Actives” dataset was submitted to the DUDE decoy online generator (Mysinger et al., 2012) to obtain decoys with similar 1D physicochemical properties but dissimilar 2D topology in comparison to the active compounds. Using this strategy, a “Decoy” set comprising 18 043 substances was created.

### Conformational sampling, ligand set clustering

The “High Actives,” “Decoys,” and “True Inactives” sets were transferred into multi-conformational databases via i:Con with the default “BEST” settings [Timeout (s): 600, RMS threshold: 0.8, energy window: 20.0, max. pool size: 4,000, max fragment build time: 30, max number of conformers: 200]. The 338 compounds of the “High Actives” set were clustered in LigandScout 4.08 using the implemented pharmacophore clustering tool. The tool clusters molecules with similar pharmacophore characteristics in the dataset: It generates pharmacophores for each molecule in the dataset for a desired number of conformations. The similarity of these pharmacophores is measured with the cosine similarity (value between 0 and 1) of their radial distribution function score (RDF) vectors. Options, distance 0.9 and cluster distance calculation method “maximum” with three conformations for each molecule, were used.

### Pharmacophore generation

With LigandScout pharmacophores can be generated as shared or merged feature pharmacophores. A shared feature pharmacophore only appoints common features observed during 3D alignment of the validation set molecules. A merged feature pharmacophore merges several shared pharmacophores. Features, which are not shared by the whole validation set, are appointed as optional. The initial pharmacophore model for cluster 7 was built as a shared feature pharmacophore with six molecules as templates using “pharmacophore fit and atom overlap” as the scoring function. The initial model for cluster 12 was built as a merged feature pharmacophore with four molecules as templates using “pharmacophore fit and atom overlap” as the scoring function. The models were refined and theoretically validated until favored theoretical performance was achieved.

### Theoretical validation

For theoretical validation, the scoring function was set to “pharmacophore-fit,” the screening mode to “match all query features” with maximum number of omitted features zero. To assess the performance of the individual models, the resulting hit list were used to calculate common enrichment metrics, as comprehensively outlined in a review by Seidel and coworkers (Seidel et al., 2010).

### Virtual screening

Several freely available molecular structure databases were deployed for VS, having a strong focus on NP. The conformational libraries were generated with i:Con (Friedrich et al., 2017). Depending on the size of the database the recommended “BEST” or “FAST” settings were used (Table 1). For VS, the same settings were used as in the theoretical validation, although the retrieval method “get best matching conformation” was used.

**Table 1:** Screened databases with content type (origin of molecules) and size (number of molecules), their source and the used standard settings for conformer generation with i:Con.

**Database**	**Number of molecules**	**Type**	**Source**	**Settings**
Analyticon-MEGx	4,355	NPs	Analyticon http://www.ac-discovery.com/	Best settings
Analyticon-Triterpenes	409	NPs	Analyticon http://www.ac-discovery.com/	Fast settings
Drugbank	6,863	Approved/ trialed drugs	DrugBank 4.5 http://www.drugbank.ca/	Fast settings
In house database Department of Pharmacognosy; University of Vienna	1,152	NPs	In house (Update 2016)	Fast settings and best settings
NPDB	115,275	NPs	(Rollinger et al., 2004)	Fast settings
NuBBE	1,628	NPs	(Valli et al., 2013) http://nubbe.iq.unesp.br/portal/ nubbedb.html	Best settings
SPECS NP	871	NPs	SPECS http://www.specs.net/	Fast settings
SPECS SC	212,446	Synthetic compounds	SPECS http://www.specs.net/	Fast settings
TCM-Taiwan	35,993	NPs	(Chen, 2011) http://tcm.cmu.edu.tw/	Fast settings

### Hit list prioritization

A principal component analysis using the chemGPS online tool (Larsson et al., 2007) was determined and a hierarchical cluster analysis with SIMCA facilitated the assignment of the compounds into 9 groups, each inhabiting a different chemical space. For clustering, the default Ward's minimum variance agglomerative clustering algorithm for the quantitative first three chemGPS principal components, PC1, PC2, and PC3, were used. A molecule's size, polarizability and shape are characterized by PC1, while PC2 describes its aromatic and conjugation-related properties and PC3 corresponds to its lipophilicity, polarity, and hydrogen bond (HB) capacity. Shape-focused VS was performed with Open Eye ROCS to retrieve a TC score, which combines a shape matching with a chemistry alignment Tanimoto score. This scoring function assesses the goodness of the alignment between the query and the candidate molecules. ComboScore puts exactly equal weights on both of its components, a shape-based scoring function and a function considering pharmacophore-like chemical pattern matching. Theoretically, the TC score can lie between 0 and 2 (Hawkins et al., 2007; OpenEye, 2016). The best fitting conformation of **13** derived from the alignment with model BAMS22 was used as query with the ROCS default options. The PAINS filters of the FAF-Drugs 4 online tool were applied to identify potential promiscuous hitters.

### *In vitro* GPBAR1 activity and statistical analysis

*In vitro* evaluation of the selected hit list was performed with a reporter gene-based luciferase assay in HEK 293T cells (obtained from ATCC, USA), which was described previously (Ladurner et al., 2017). Cells were grown and maintained in Dulbecco's modified eagle medium (DMEM) without phenol red with 10% heat-inactivated fetal bovine serum (FBS), 4.5 g/L glucose, 2 mM glutamine, 100 U/mL benzylpenicillin, and 100 μg/ml streptomycin. During the experiments, charcoal-stripped medium with 5% FBS was used. 6 × 10^6^ cells were grown in 15 cm dishes for 19 h and then transiently transfected using the calcium phosphate method with 5 μg of a GPBAR1 expression plasmid and 5 μg of a CRE-Luc plasmid. For later normalization, 3 μg of an EGFP expression plasmid was co-transfected. Control experiments were performed with cells transfected only with 3 μg EGFP and 5 μg CRE-Luc plasmids. After 6 h, transfected cells were reseeded to 96 well plates (5 × 10^4^ cells/well) and incubated with 5 μM and 20 μM compound dilutions, respectively, for 18 h. 0.1% DMSO served as vehicle control and 10 μM LCA as positive control. After incubation the medium was removed, and the plates were immediately frozen at −80°C. Plates were kept frozen for at least 1 h to facilitate lysis and measurements were performed in the following 10 days. For the measurement, cells were thawed, lysed and transferred to black 96-well plates. After addition of ATP and luciferin, emitted luminescence and fluorescence was measured with a Tecan Infinite 200 PRO plate reader (Tecan, Austria). GPBAR1 activity was expressed as fold activation compared with the solvent control (0.1% DMSO) or as % activation compared to the positive control 10 μM LCA (arbitrary 100% activation). The measured relative luciferase units (nRLU) were normalized to the transfected cell mass expressed as EGFP-derived relative fluorescence units (RFU) from at least three independent experiments (mean values ± standard error mean) performed in quadruplicate. Quantified EGFP-derived fluorescence was used as an indicator for transfected cell mass and thus used to assess the compounds' cytotoxicity. Compounds, which resulted in significantly lower RFU values than the control, were considered as cytotoxic. For statistical analysis GraphPad Prism 4.03 was used. Statistical significance was assessed by One Way ANOVA and Bonferroni post-test (^***^p < 0.001, ^**^p < 0.01, ^*^p < 0.05, ns not significant). Non-linear regression was used to calculate EC_50_ values with the sigmoidal dose response (variable slope) settings.

### Compounds and chemicals

Hederagenin (CAS#465-99-6) and bayogenin (CAS#80368) were ordered from Phytolab (Germany). 2,3-O-isopropylidenyl-euscaphic acid (CAS#220880-90-0) was ordered from Proactive Molecular Research (USA). Phytolaccoside B (CAS#60820-94-2) and euscaphic acid (CAS#53155-25-2) was purchased from Cambridge Chemicals (USA). Phytolaccagenic acid (CAS#54928-05-1) and 16-dehydropregnenolone (CAS#1162-53-4) were obtained from Carbosynth (UK). Spironolactone (CAS#52-01-7) and methylhyoxycholate (CAS#2868-48-6) were purchased from TCI Deutschland GmbH (Germany). The screening compounds (CAS#1019061-83-6, CAS#303139-94-8, CAS#330636-58-3, CAS#353253-76-6, CAS#353779-79-0, CAS#432530-00-2, CAS#444931-63-9, CAS#496937-29-2, CAS#791840-52-3, CAS#902244-06-8, CAS#915930-57-3, CAS#932954-51-3, CAS#352644-32-7, CAS#500218-51-9, CAS#314757-83-0, CAS#380633-89-6, CAS#26179-09-9, CAS#664993-86-6, CAS#525577-20-2) were obtained from SPECS (Netherlands). Microlobidene (CAS#89783-66-4) and farnesiferol B (CAS#54990-68-0) were available from a previous project (Rollinger et al., 2008). Nordihydroguaretic acid (CAS#500-38-9) was purchased from Fluka (Switzerland). The positive controls LCA (CAS#434-13-9) and CDCA (CAS#474-25-9) were obtained from Sigma Aldrich (Austria). Alphitolic acid (CAS#19533-92-7), was obtained by hydrolysis from a previously isolated saponin (Mair et al., 2018). The purity was checked using UPLC-PDA-MS and determined as ≥ 98% for compounds **20**, **21**, **23**, **24**, **28**, **30**-**35**, **37**, **40**, **41**, **44**-**46**, **48**, **49**, and **52**. For all other compounds it was between 90 and 98%. MS and NMR data of all in house compounds (**27**, **28**, **52**) are provided in the literature (Rollinger et al., 2008; Mair et al., 2018) and the Supplementary Information (Supplementary Figures 4–10).

### Cell culture reagents and plasmids

DMEM, L-glutamine, benzylpenicillin and streptomycin were purchased from Lonza, (Switzerland), FBS, and trypsin were obtained from Gibco via Invitrogen (Austria). The GPBAR1 transcript variant 3 (NM170699) plasmid was obtained from Origene via Biomedica (Vienna, Austria). The CRE-Luc plasmid, (pGL4.29[luc2P/CRE/Hygro), luciferase assay system and used lysis buffer were ordered from Promega (Germany) and the EGFP (pEGFP-N1) plasmid was purchased from Clontech (USA).

## Results and discussion

### Workflow

**Figure 2:**
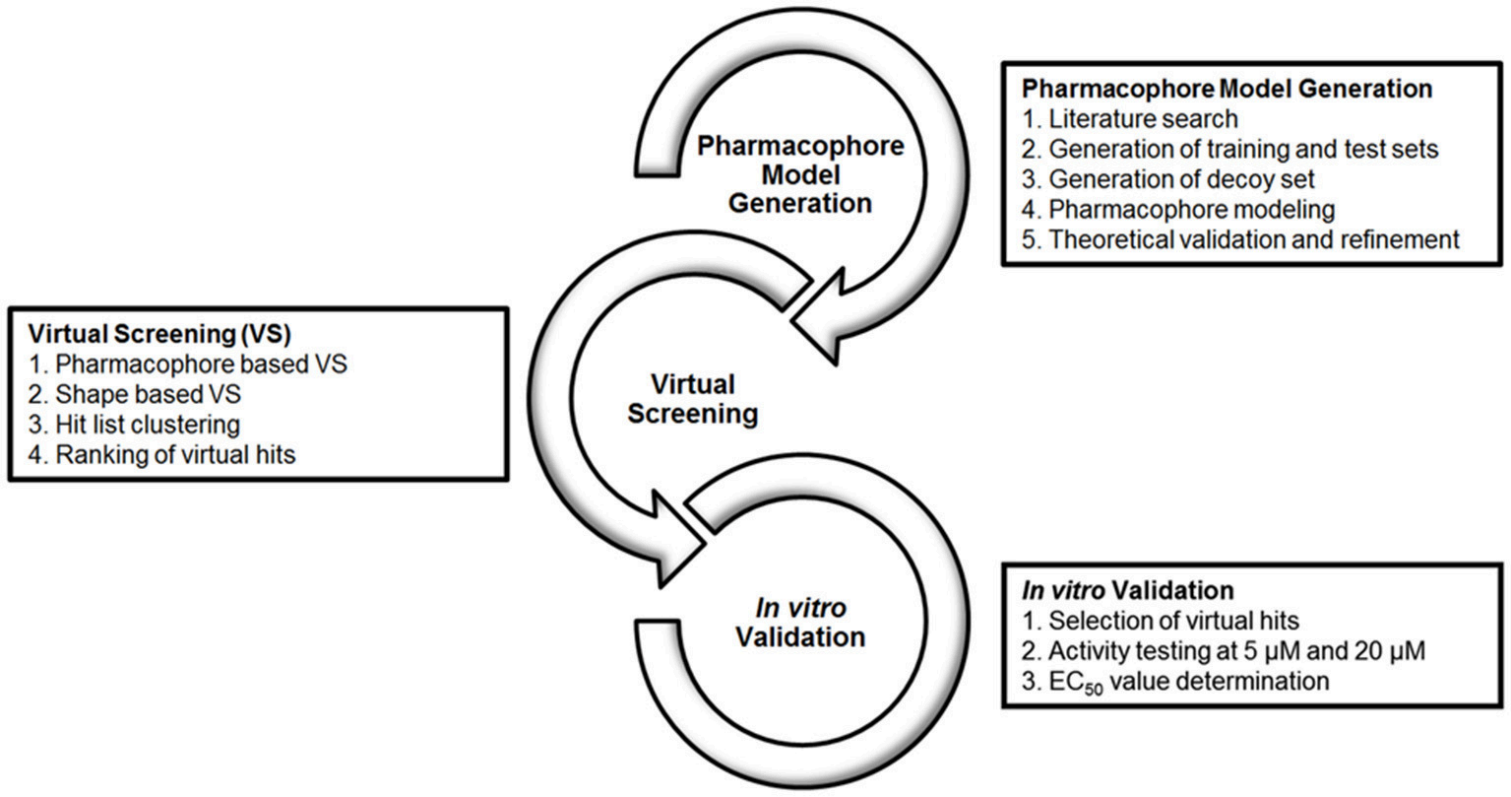
Workflow of model generation, VS and experimental validation.

The workflow of this study is divided into 3 levels, as depicted in Figure 2: (1) Literature search for the compilation of a database of known GPBAR1 actives and inactives to be split and used as pharmacophore training set and a validation set for theoretical validation; The generation of a pharmacophore model collection with LigandScout and the subsequent theoretical validation. (2) VS of multi-conformational databases consisting of structures of natural and synthetic compounds (Table 1) using the two most promising models as queries; Evaluation of the hit list applying shape-based screening and physicochemical space clustering of virtual hits. (3) Selection of 33 virtual hits and their experimental validation in a HEK 293T cell based luciferase assay.

### Pharmacophore modeling

**Figure 3:**
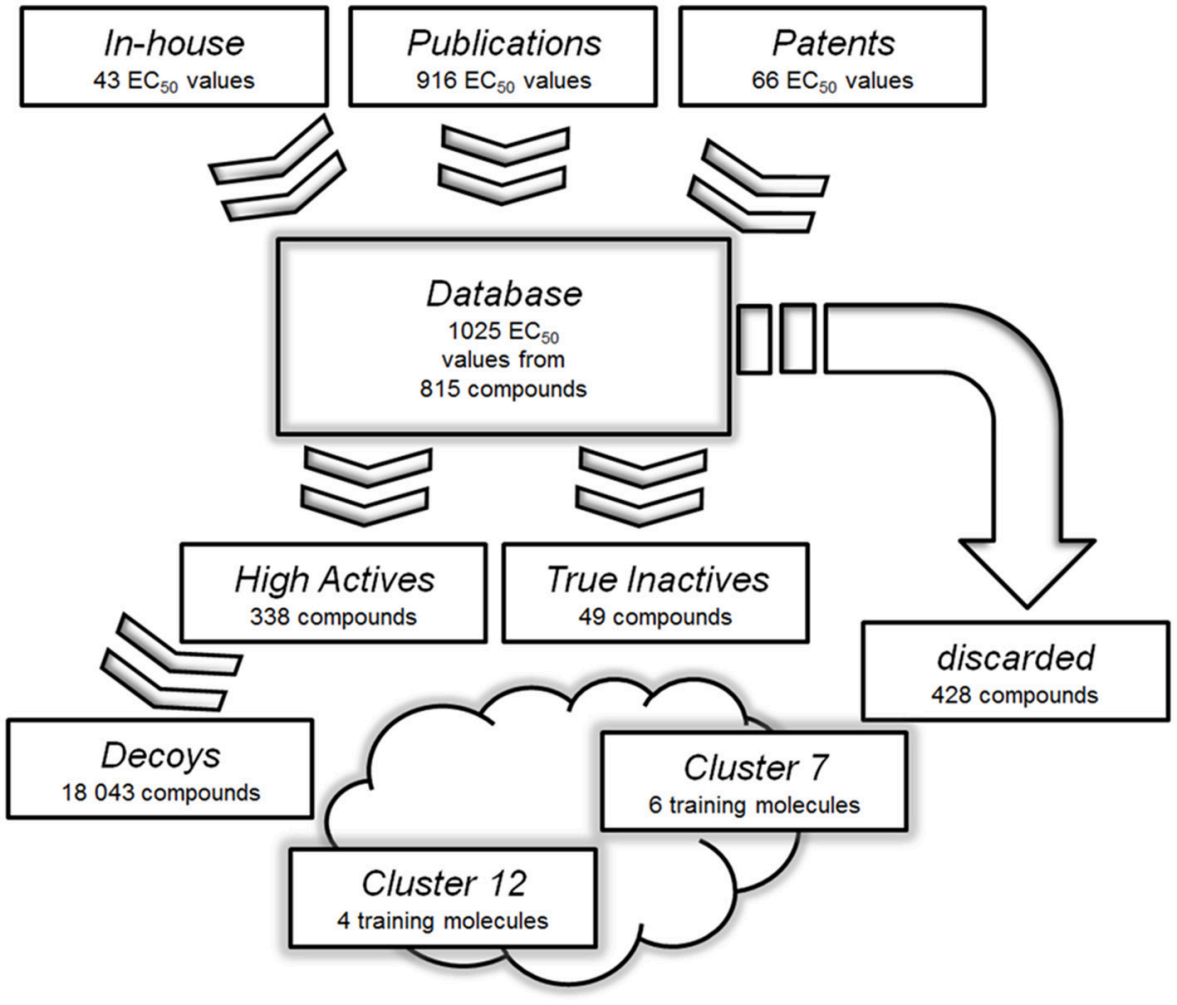
Databases used for the generation and validation of the pharmacophore models: Molecules with reported or measured GPBAR1 agonistic activity were gathered into a comprehensive “Database”. Compounds with low or not assignable bioactivities were discarded. The “High Actives” dataset was grouped into 12 clusters using LigandScout's pharmacophore clustering tool. The two best-performing models were derived based on training sets from clusters 7 and 12. The clusters were further used to generate validation sets for corresponding models.

A pharmacophore model is the abstract three dimensional representation of the molecular interactions between a target and a ligand structure, which is reduced to a collection of steric and chemical features that are necessary to trigger a desired pharmacological effect. The quality of a ligand-based pharmacophore model strongly relies on the selection of training set molecules. Therefore, it is mandatory to strictly select only highly potent activators for the training and validation sets (Seidel et al., 2010). In the case of GPBAR1, available bioactivity data were not only obtained by different working groups, but also with different cellular assays. This raised concerns about direct data comparison among the used assays. Only ligands tested clinically or with a reported activity, which was proven to be both potent and directly comparable to respective positive controls, were therefore used in this study. Subsequently, 428 of 815 compounds had to be discarded. The remaining 338 compounds formed the “High Actives” dataset. 49 compounds were categorized as “True Inactives.” The data handling used as basis for the generation of the ligand-based phamacophore models is illustrated in Figure 3.

A pharmacophore model built of several query compounds binding at different ligand binding sites to the target protein would clearly distort the quality of such a model and devastate its predictive power. Therefore, the “High Actives” dataset compounds were divided into 12 clusters using the pharmacophore clustering tool implemented in LigandScout. Cluster 1 was discarded as it only consisted of one compound. The remaining 11 clusters contained between 11 and 80 molecules and were separated each into test and validation set.

**Figure 4:**
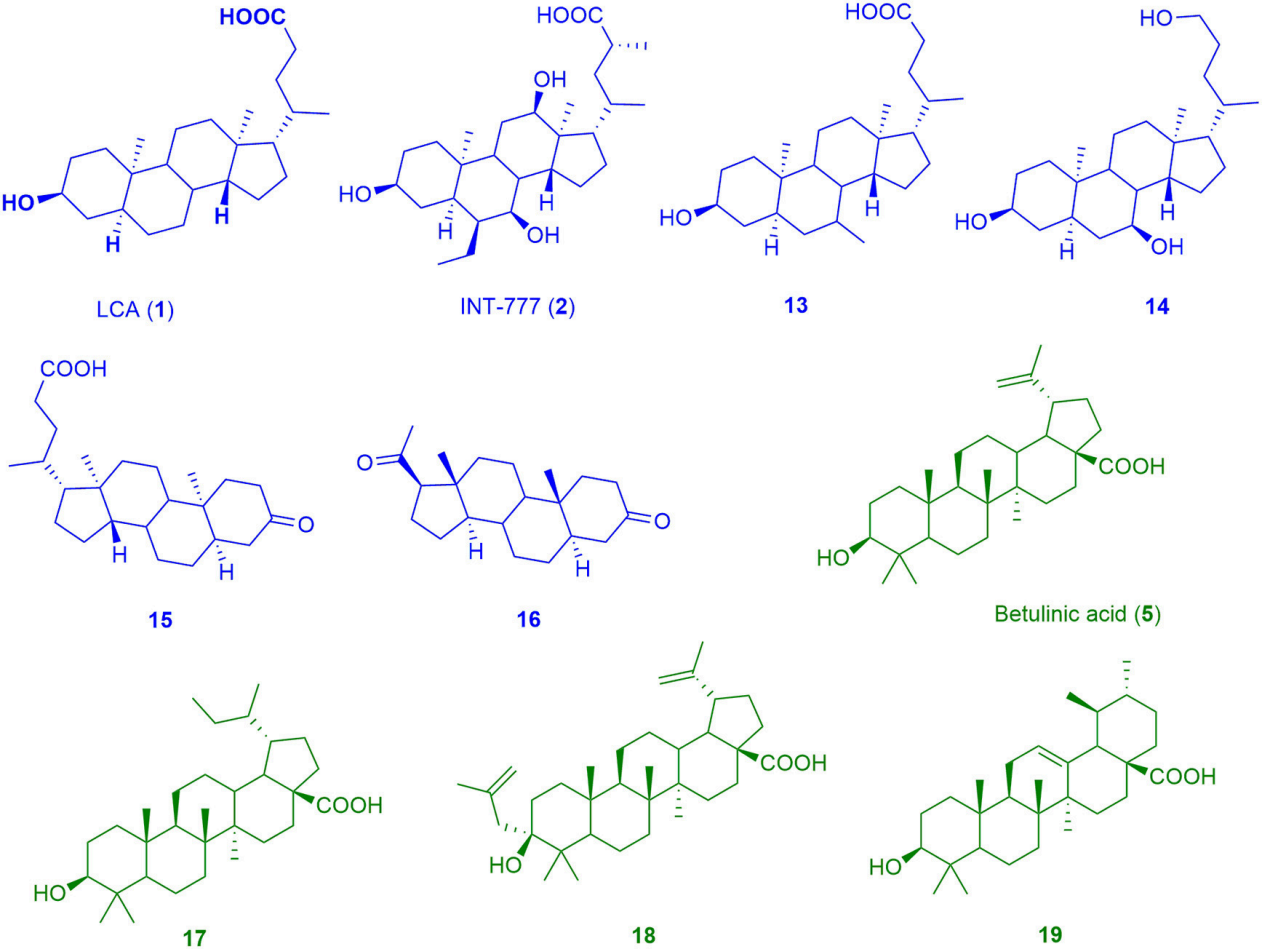
Chemical structures of training compounds for the generation of the pharmacophores BAMS22 (blue molecules) and TTM8 (green molecules).

Out of the retrieved 11 cluster sets, 12 pharmacophore models were generated. Altogether, in parallel screening, these models were able to predict 275 of 338 compounds (81%) in the “High Actives” database as true positives. However, a high number of false positives were retrieved, when the models were screened against the “Decoys” and “True Inactives” databases. This resulted in poor metrics of this entire model collection's enrichment factor (*EF* = 11.22). Two models, which were based on the pharmacophores of natural products, showed promising metrics and were selected for the prospective VS and experimental validation. The first model, BAMS22, was based on a training set of 6 molecules (depicted in Figure 4) resulting from cluster 7. They had been selected for covering nearly the whole physicochemical space and for incorporating most of the structure-activity information contained in cluster 7. BAMS22 was used for VS of the “Decoys”/“True Inactives” (*n* = 18.112) databases and the cluster 7 validation set (*n* = 20), which resulted in a specificity of 1 (0.998785) and a sensitivity of 1, achieving an EF of 823.3. Along with the molecules from cluster 7, the potent ligand TLC from cluster 12 was retrieved as highly ranked virtual hit.

**Figure 5:**
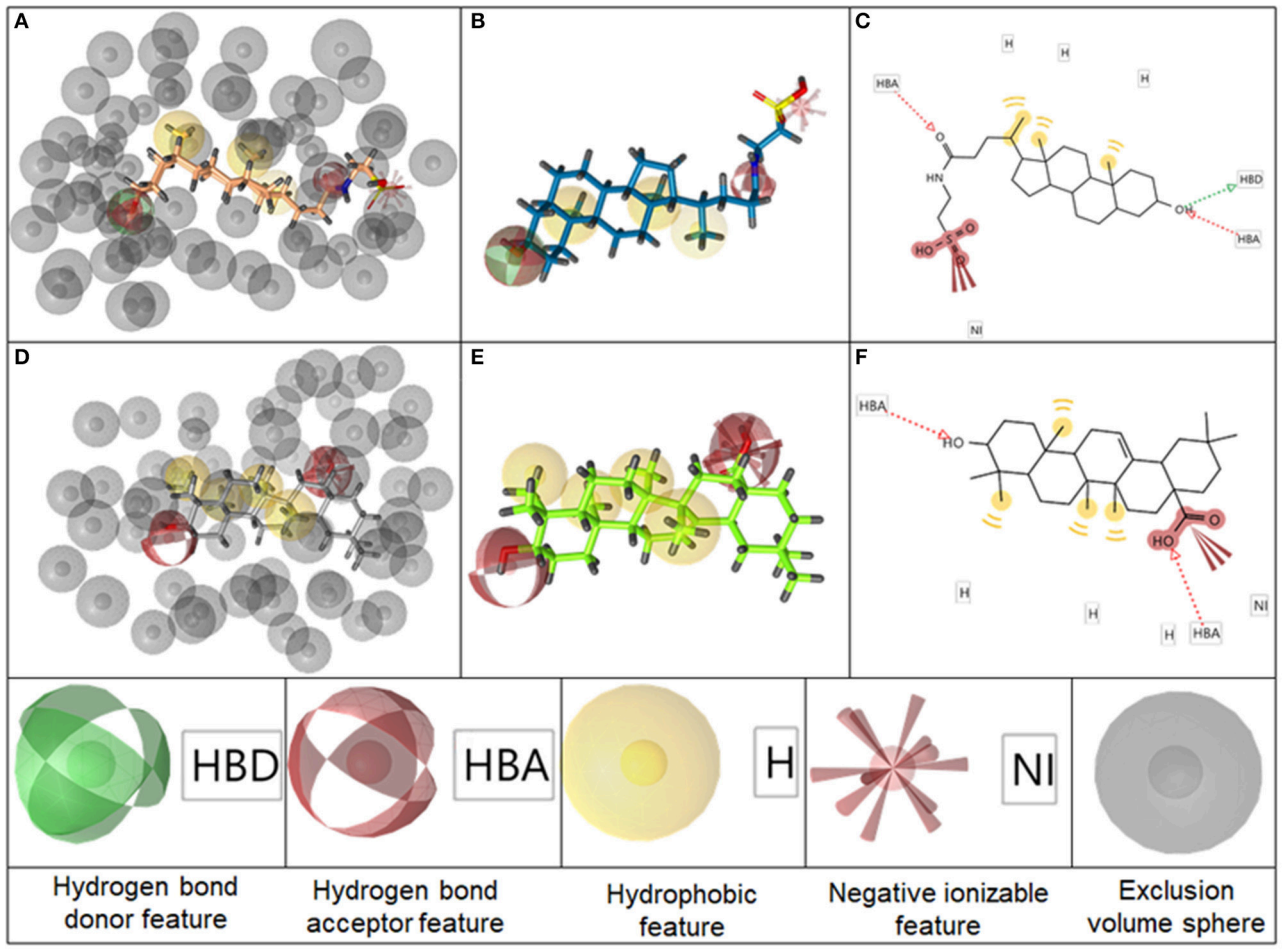
Representation of the pharmacophore model BAMS22 aligned to TLC in 3D with exclusion volume spheres **(A)**, without exclusion volumes **(B)** and in 2D **(C)**. Depiction of TTM8 aligned to oleanolic acid (**6**) in 3D with exclusion volume spheres **(D)**, without exclusion volumes **(E)** and in 2D **(F)** The gray spheres in **A,D** depict so-called exclusion volumes reflecting steric hindrances. The colored spheres represent the pharmacophore features, explained at the bottom, whereby opaque spheres represent mandatory features and spheres with light shading optional ones. In the 2D graphs **(C,F)**, HB features are illustrated as dashed arrows, hydrophobic features as yellow circles and negatively ionizable features as red marks with red bolts attached.

The BAMS22 model consists of two mandatory hydrophobic features, two mandatory HB acceptor features, an optional HB donor, an optional hydrophobic, and an optional negatively ionizable feature, as well as a rigid exclusion volume coat. In agreement to the TLC binding predictions and experiments of Gertzen and coworkers (Gertzen et al., 2015), our pharmacophore model, although not based on the homology model's input information, depicts a very similar interaction pattern (Figure 5). Gertzen stated that the 3-hydroxyl moiety of TLC forms a HB to E169 and Y240, the sulfonic acid group forms a salt-bridge to R79 and hydrophobic interactions appear with L244. All of these statements were underlined with alanine-scanning experiments and are in accordance with our model. The model also suggests a second important HB interaction with the C-24 carboxamide group of TLC (Figure 5C), as well as with the C-24 hydroxyl group of **14**, or in the case of **16** with the C-20 keto group. The hydrophobic interactions were placed where hydrophobic alignment was possible. Although the model showed a very high specificity, it only consisted of four mandatory pharmacophore features, two HB accepting and two hydrophobic features, a widespread pattern of pharmacophore features. Therefore, not only steroid-like structures can putatively be retrieved in the prospective VS.

It is questionable whether triterpenes and bile acids share the same binding mode, as it was not possible to generate a restrictive pharmacophore model incorporating both scaffolds, although the binding modes appear to be very similar. It is likely that they have a different binding mode within the same binding position. Therefore, it was preferred to explain the steroidal structures with two highly specific local models and not with a single global model. It has previously been acknowledged for the identification of cyclooxygenase inhibitors that a set of highly specific local models leads to lower false positive hit rates, compared to one pleiotropic global model (Schuster et al., 2010).

The second model, TTM8 is based on a training set of 4 molecules (Figure 4) from cluster 12. The model consists of 4 mandatory hydrophobic, two mandatory HB acceptor features, and a mandatory negatively ionizable feature (Figure 5). TTM8 was theoretically validated against the set of “Decoys”/”True Inactives” datasets (*n* = 18,112) and the cluster 12 validation set (*n* = 16), and showed a specificity of 1 and sensitivity of 0.81, achieving an EF of 919.5.

Genet and co-workers (Genet et al., 2010) were the first evaluating the SAR of triterpenes on the GPBAR1 receptor. They concluded that essential features for agonistic activity are a 3α-hydroxyl group, a carboxyl group in position 17α, and a rigid pentacyclic scaffold, in the best case a lupane backbone with high lipophilicity. Further publications regarding triterpenes are scarce, although some have shown higher selectivity over FXR and higher potency on the GPBAR1 than bile acids. Therefore, a cherry-picking pharmacophore model, highly sensitive to these pentacyclic triterpene acids, was created. It can be considered as a highly suitable filtering tool with a high applicability in *in silico* assisted NP research as previously reported e.g., for pharmacological profiling of secondary metabolites or target identification of NPs (Schuster, 2010; Waltenberger et al., 2011; Grienke et al., 2015; Kratz et al., 2016).

### Prospective virtual screening and hit selection

**Figure 6:**
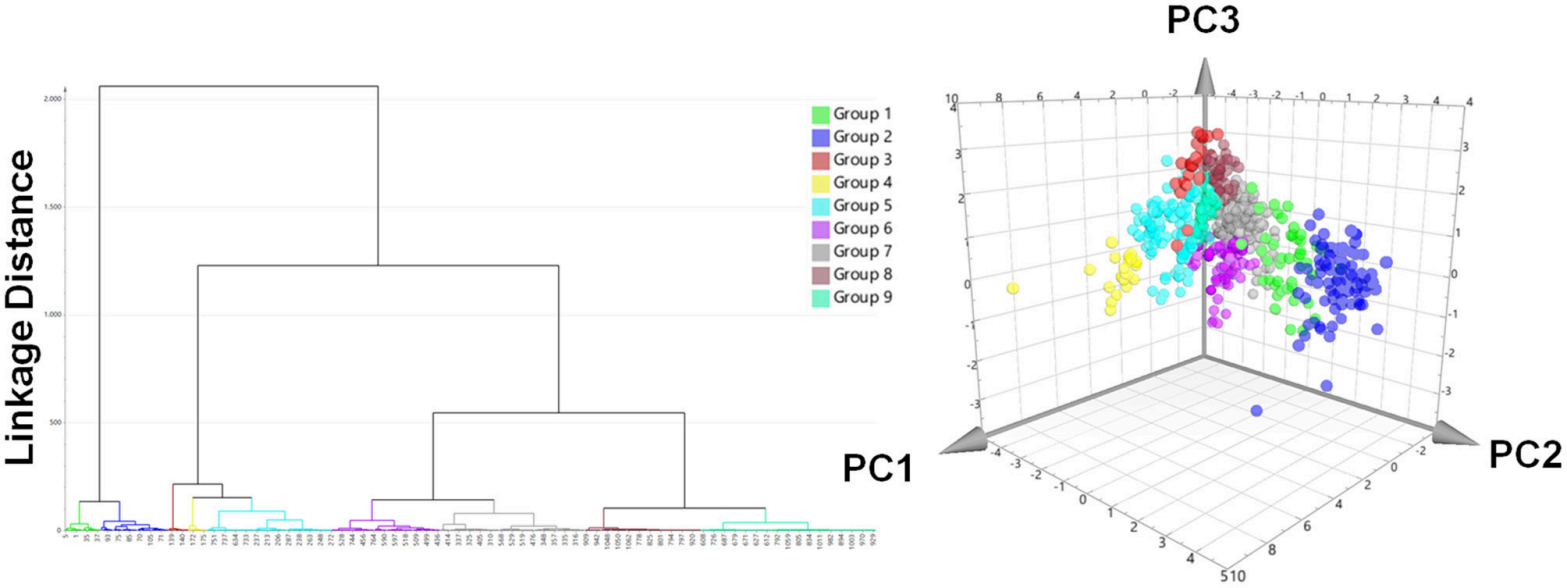
Hit list clustering into 9 groups and chemical space analysis of virtual hits. Left: Hierachical cluster analysis dendrogram with Ward's clustering technique using the first three chemGPS (Larsson et al., 2007) calculated principal components (PC1, size; PC2, aromaticity; PC3, lipophilicity) as parameters. The linkage distances were expressed as Euclidean distances. The height of the branches is similar to the distance of each node. Right: The chemical space analysis of the hit list with molecules colored according to their groups. The position of each virtual hit is defined by its calculated chemGPS principal components.

A prospective VS was performed with the two pharmacophores against over 350,000 molecules from nine different databases (Table 1). After removing duplicates, 1,069 virtual hits were obtained and clustered according to physicochemical diversity into 9 groups (Figure 6). As obvious from Figure 6, groups 1 and 2 differ from the other groups on a very early hierarchical level. The main structural difference of these two groups compared to the others is that they comprise synthetic compounds and NPs with aromatic rings, more conjugated double bonds and heteroatoms, while groups 3–9 consist of steroidal structures, reaching from cardenolides, pregnanes, bile acids to steroids and triterpenes. The most interesting molecules, in terms of structural diversity, are found in groups 1 and 2, as they comprise scaffolds dissimilar to the query molecules of the underlying pharmacophore models.

For prioritization of virtual hits to be experimentally tested a ranking was performed using shape-focused VS employing the ROCS Tanimoto Combo (TC) score (Hawkins et al., 2007; OpenEye, 2016). For this purpose best matching conformations derived from the pharmacophore-based VS were aligned with query molecule **13**. Hit selection considered a high TC score, but also compound availability in sufficient purity, and structural variance. Finally, 33 compounds were subjected to experimental validation (Table 2): 11 compounds had been clustered in groups 4–9 (Supplementary Figure 3), 9 compounds in group 1 (Supplementary Figure 1), and 13 compounds in group 2 (Supplementary Figure 2).

**Table 2:** Results of the experimental validation of the virtual hits tested at 5 μM and 20 μM, the pharmacophore fit score (PF), the TC score (calculated in OpenEye ROCS with **13** as a query), the model with which they were predicted and their underlying database.

**Compound**	**Database**	**predicted by BAMS22**	**predicted by TTM8**	**PF score**	**TC score**	**Activity (at 5 μM)**	**Activity (at 20 μM)**
**20**	SPECs	**X**		45.31	0.873	2.34% (± 1.40)	1.74% (± 1.36)
**21**	SPECs	**X**		56.55	0.913	Not evaluable neither at 5 μM nor 20 μM, due to cytotoxic properties	
**22**	SPECs	**X**		46.23	0.703	1.42% (± 1.39)	−0.06% (± 0.39)
**23**	SPECs	**X**		56.31	0.720	1.69% (± 1.01)	11.00% (± 4.15)
**24**	SPECs	**X**		46.23	0.703	4.75% (± 1.51)	6.12% (± 2.01)
**25**	SPECs	**X**		45.24	0.627	1.16% (± 0.35)	0.70% (± 0.54)
**26**	SPECs	**X**		45.08	0.701	0.47% (± 1.82)	0.58% (± 0.89)
**27**	In house	**X**		46.39	0.737	4.70% (± 1.99)	60.85% (± 20.05)
**28**	In house	**X**		56.72	0.726	5.07% (± 1.36)	83.81% (± 12.00)
**29**	SPECs	**X**		46.23	0.783	1.54% (± 0.91)	1.65% (± 0.69)
**30**	SPECs	**X**		47.83	0.860	−0.60% (± 0.36)	0.92% (± 0.67)
**31**	SPECs	**X**		46.32	0.806	−0.31% (± 0.38)	0.88% (± 1.69)
**32**	SPECs	**X**		57.09	0.877	3.00% (± 0.58)	19.45% (± 6.00)
**33**	SPECs	**X**		56.50	0.801	0.98% (± 1.13)	1.00% (± 0.18)
**34**	SPECs	**X**		65.59	0.773	−0.02% (± 0.14)	9.27% (± 2.62)
**35**	SPECs	**X**		46.2	0.777	−0.06% (± 0.44)	3.10% (± 0.46)
**36**	SPECs	**X**		46.15	0.821	1.07% (± 0.10)	0.34% (± 0.18)
**37**	SPECs	**X**		46.28	0.838	0.33% (± 0.63)	3.87% (± 1.89)
**38**	SPECs	**X**		45,25	0.810	1.67% (± 2.54)	Not evaluable at 20 μM, due to cytotoxic properties
**39**	SPECs	**X**		46.14	0.774	2.63% (± 0.69)	2.45% (± 1.42)
**40**	In house	**X**		46.16	0.783	−0.13 (± 0.31)	1.72% (± 0.90)
**41**	NPDB	**X**		65.87	0.664	3.73% (± 2.99)	4.35% (± 1.28)
**42**	TCM-DB		**X**	73.71	0.662	3.76% (± 1.67)	8.78% (± 3.87)
**43**	NPDB		**X**	73.16	0.815	3.23% (± 0.86)	26.28% (± 2.18)
**44**	NPDB		**X**	73.41	0.779	3.45% (± 4.66)	22.19% (± 15)
**45**	NPDB	**X**		55.93	1.222	5.20% (± 2.42)	22.58% (± 0.74)
**46**	drugbank	**X**		46.71	0.938	12.80% (± 6.60)	Not evaluable at 20 μM, due to cytotoxic properties; at 15 μM: 17.34% (± 0.91)
**47**	NPDB	**X**		56.19	0.954	Not evaluable neither at 5 μM nor 20 μM, due to cytotoxic properties	
**48**	NPDB	**X**		68.54	1.611	1.13% (± 0.77)	23.22% (± 5.29)
**49**	In house		**X**	72.75	0.896	1.99% (± 1.07)	12.08% (± 6.51)
**50**	In house		**X**	73.53	0.897	12.69% (± 5.33)	35.95% (± 2.37)
**51**	In house		**X**	72.75	0.864	−1.39% (± 1.17)	Not evaluable at 20 μM, due to cytotoxic properties
**52**	In house		**X**	73.88	0.822	29.79% (± 10.79)	Not evaluable at 20 μM, due to cytotoxic properties
Vehicle control (0.1% DMSO)						0%	
LCA (**1**) (10 μM)						100%	
CDCA (**3**) (50 μM)						66.69% (± 8.72)	

### Biological evaluation

GPBAR1 activity of selected hits (Supplementary Figures 1–3) was determined in a reporter gene-based luciferase assay performed in HEK 293T cells. This assay assesses the upregulation of the cAMP-PKA-CREB pathway upon GPBAR1 activation and all conclusions are therefore limited to this receptor pathway. Compounds were considered active when they achieved at least 50% receptor activation. Compounds reaching at least 15% receptor activation were counted as weak activators. The response to 10 μM LCA was set to 100% receptor activation. Vehicle control with a final dimethylsulfoxide (DMSO) concentration of 0.1% was set to 0% activation. Initially, compounds were tested at 2 concentrations, i.e., 5 μM and 20 μM. From the 33 compounds, only two (**47** and **50**) were cytotoxic in both concentrations tested. From the remaining 31 compounds, two showed significant activity with more than 50% receptor activation at 20 μM and six further compounds achieved more than 15% receptor activation either at 5 μM or 20 μM (Table 2). Compounds **22**, **24**, **30**, **34-36**, and **41** were identified as potential pan-assay interference substance (PAINS) but none of them showed activity in the experimental validation. At 5 μM, only one compound (**52**) achieved the arbitrary threshold of 15% receptor activation. Spironolactone (**46**) an approved drug for the treatment of heart failure, showed 17.3 % receptor activation at 15 μM. Table 2 gives an overview of the experimental results.

**Figure 7:**
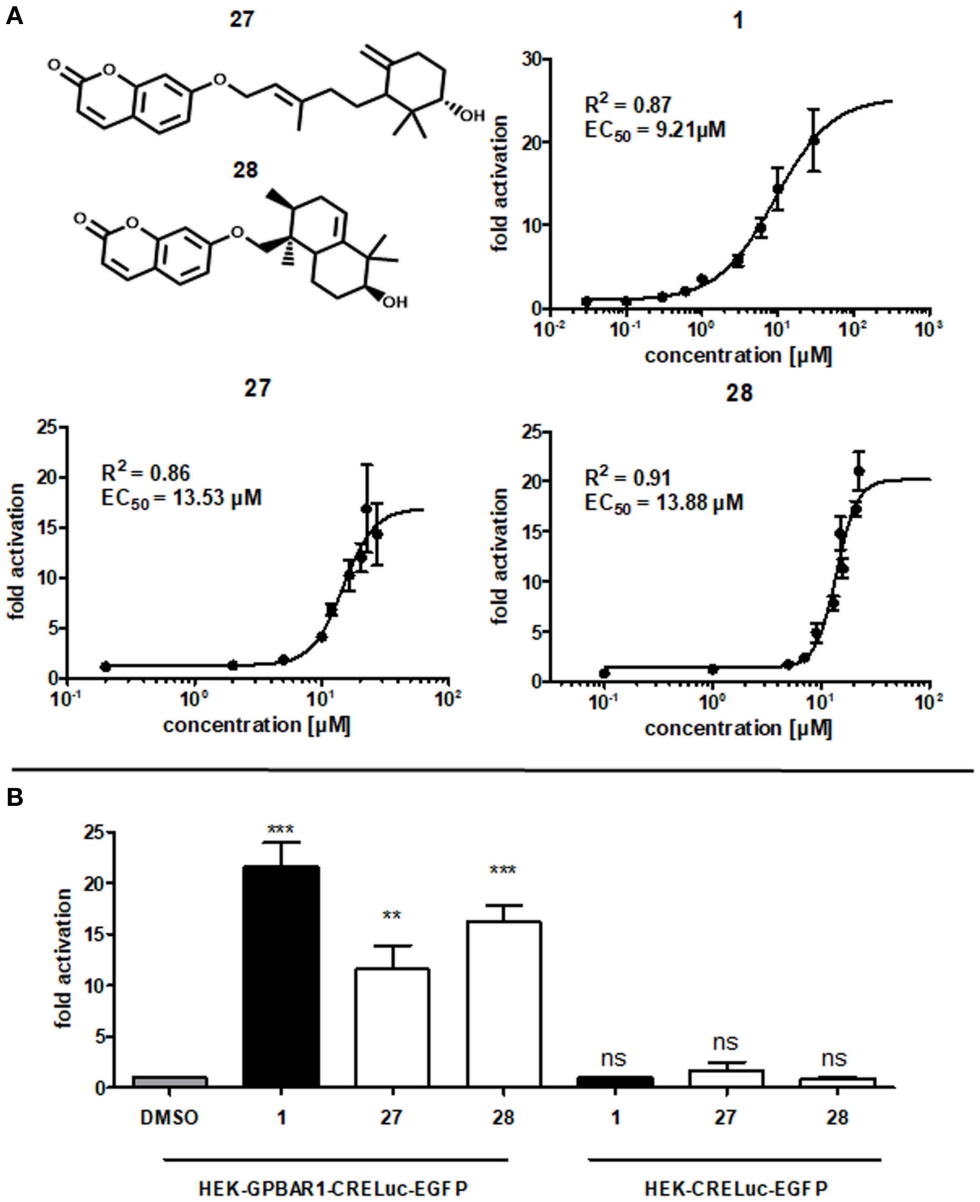
**(A)** Concentration-dependent increase of GPBAR1 activity in response to LCA (**1**), farnesiferol B (**27**) and microlobidene (**28**). HEK-293 cells were transfected and stimulated as described in the Methods section. Luciferase activity was normalized to EGFP-derived fluorescence. Results are expressed as fold induction compared with the solvent control (DMSO, 0.1%) as the mean with SEM of at least three independent experiments. The two highest concentrations of **27** are the mean of two independent experiments. GraphPad Prism's non-linear regression with the sigmoidal dose response settings (variable slope) was used to calculate curves. **(B)** Fold activation of compounds **1** (10 μM), **27** and **28** (20 μM) in comparison to vehicle control 0.1% DMSO in (left) GPBAR1 transfected cells and (right) GPBAR1 untransfected cells. HEK 293T cells were transfected with GPBAR1, EGFP and CRE-Luc expression plasmids (left), or with EGFP and CRE-Luc expression plasmids only (right). Cells were treated for 18 h with 20 μM of **27** and **28** as well as 10 μM LCA (**1**) as positive control and 0.1% DMSO as vehicle control. Luciferase activity was normalized to EGFP-derived fluorescence. Results are expressed as fold induction compared with the solvent control (0.1% DMSO). All given values are the mean of at least 3 independent experiments and the variance is given as SEM. Significance was evaluated with one-way ANOVA-Bonferoni post-test (^***^p < 0.001; ^**^p, < 0.01; ns, not significant vs. vehicle control).

As a result of this screening, the sesquiterpene coumarins **27** and **28** were discovered to be potent activators of the GPBAR1 receptor, which corroborated the scaffold-hopping competence of BAMS22. The two compounds are present in the gum resin of *Ferula assa-foetida* L., used in central Asia as spice and medicine. The concentration response curves for **27** and **28** were determined and are shown in Figure 7. Compounds **27** and **28** were cytotoxic in transfected HEK cells at concentrations higher than 27.5 and 22.5 μM, respectively. Due to this limitation the determination of E_max_ values could not be accurately determined in this assay. Accordingly, the analyzed fold activations and extrapolated EC_50_ values of **27** and **28** are limited to the non-cytotoxic concentration-response range and may not be completely accurate. Although limited by these constraints, farnesiferol B (**27**) showed 10.54 ± 2.25 fold activation at 20 μM (60.85% ± 20.05) and an EC_50_ of 13.53 μM. Microlobidene (**28**) achieved 16.21 ± 1.64 fold activation at 20 μM (83.81% ± 12.00) and an EC_50_ of 13.88 μM. Whether the differences in sigmoidal slopes of LCA (**1**) and the newly identified ligands **27** and **28** are due to different interaction modes warrants further investigations. In the same assay the endogenous ligand CDCA only reached a fold activation of 11 ± 1.05 at 50 μM. The positive control LCA (**1**) reached 18.59 ± 0.97 fold activation at 10 μM and 20.19 ± 3.77 at 30 μM. The activity of compounds **27** and **28** can therefore be regarded as in the range of endogenous bile acids.

Many NPs are well-known PAINS or frequent hitters (Baell, 2016). In order to prevent such unintentional false-positive results, the experiments with the two GPBAR1-activating NPs have been repeated without transfecting GPBAR1. EGFP and CRE-Luc plasmids have been transfected as usual with the same concentrations. In these control experiments, none of the compounds showed a significant increase in luminescence values. In contrast to that, the increase in luminescence in GPBAR1 transfected cells in response to the positive control LCA (**1**), as well as to compounds **27** and **28**, was significant, confirming a direct interaction with GPBAR1 (Figure 7).

## Conclusion

The two presented 3D pharmacophore models have proven their quality as VS queries, both theoretically and experimentally. The combined computational and experimental efforts led to the successful identification of novel GPBAR1 agonists with unreported scaffolds derived both from nature (**27** and **28**) and from synthetic origin (**32**). They not only enlarge the chemical diversity of receptor activators, but can also be promising starting points for SAR and further optimization. It is also the first study reporting the activity of spironolactone (**46**) on GPBAR1, highlighting the possibility that already approved drugs may interact with GPBAR1. The elucidation of the mechanism underlying the GPBAR1 activation by these compounds may be an interesting starting point for further research. The physicochemical clustering process enabled a scaffold rich hit selection and a solid predictive power, with 6.5% correctly predicted strong activators and 18.8% weak activators, recommending the presented workflow for future works. The study shows that the two models in combination are qualified for their application in the future assessments of a molecules' GPBAR1 activating profile, in particular for the assessment of NPs, as the models comprise scaffolds that are widespread in nature. This is particularly helpful for increasing our insight into the molecular mechanism of traditionally used herbal remedies with complex compositions of secondary metabolites. A fast appraisal of their pharmacological profile can give direction and fast-forward research (i.e., pinpointing most promising constituents), alongside reducing expenses.

## Author contributions

JR planned and supervised the study. BK and JK created the pharmacophore models under supervision of TL and JR. BK and JK performed the virtual screening and hit selection along with UG. BK and AL conducted biological experiments and analyzed the data under supervision of VD. The manuscript was written with contributions of all authors. All authors have given approval to the final version of the manuscript.

### Conflict of interest statement

The authors declare that the research was conducted in the absence of any commercial or financial relationships that could be construed as a potential conflict of interest.
