# Supplementary material for: In Silico Workflow for the Discovery of Natural Products Activating the G Protein-Coupled Bile Acid Receptor 1
Source: Front Chem. 2018 Jul 2;6:242. doi: 10.3389/fchem.2018.00242 (PMC6036132; doi:10.3389/fchem.2018.00242)
Supplement: Supplementary file 1 [file Data_Sheet_1.pdf]

## *Supplementary Material*

# IN SILICO WORKFLOW FOR THE DISCOVERY OF NATURAL PRODUCTS ACTIVATING THE G PROTEIN-COUPLED BILE ACID RECEPTOR 1

**Benjamin Kirchweger, Jadel M. Kratz, Angela Ladurner, Ulrike Grienke, Thierry Langer, Verena M. Dirsch, Judith M. Rollinger***

*** Correspondence:** judith.rollinger@univie.ac.at

## 1 Supplementary Figures

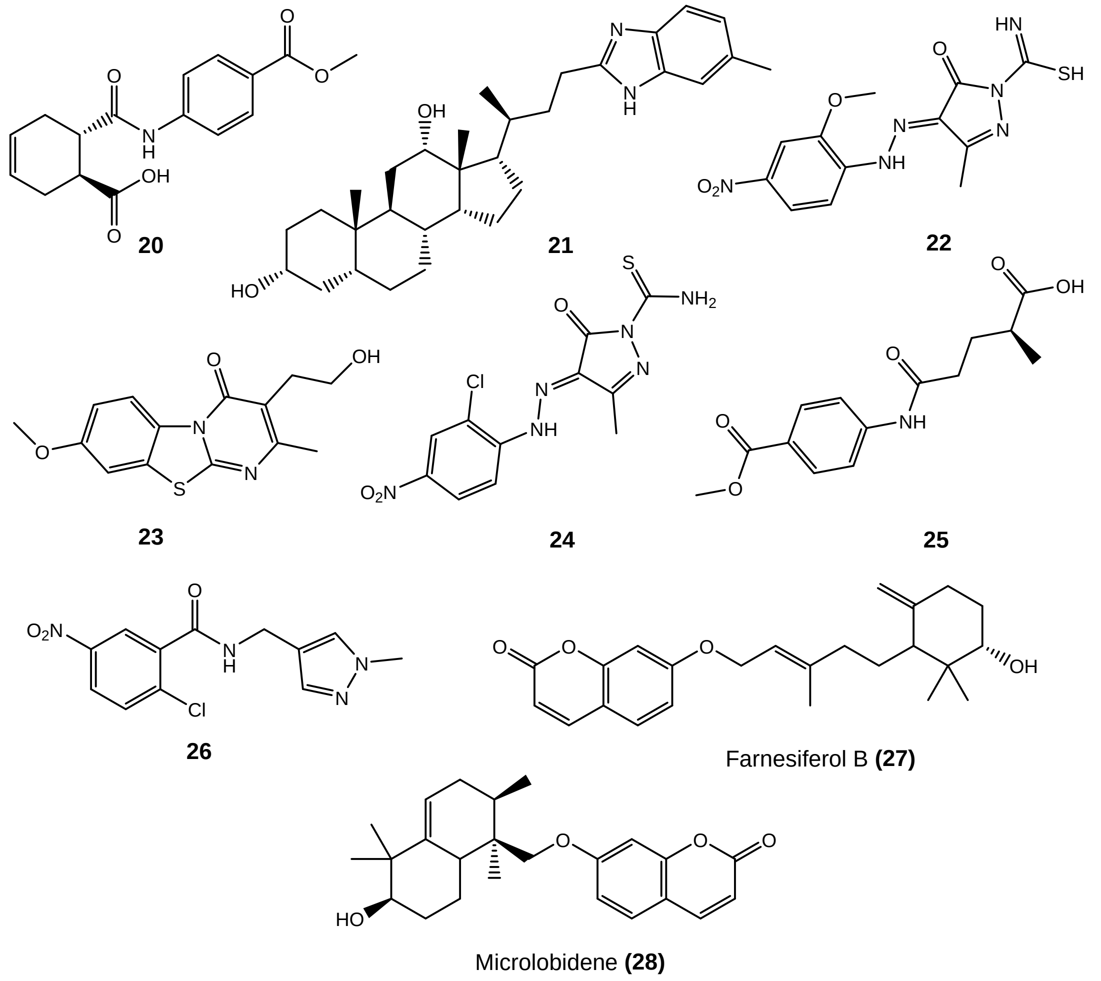


**Supplementary Figure 1.** Chemical structures of selected virtual hits from group 1; farnesiferol B (**27**) and microlobidene (**28**) are secondary plant metabolites, **20-26** are synthetic compounds obtained from SPECs.

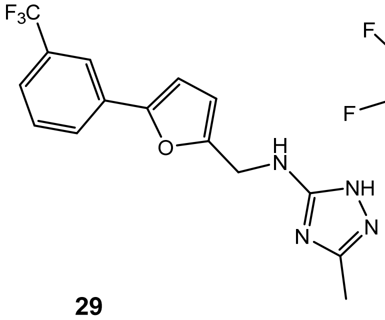
**29**

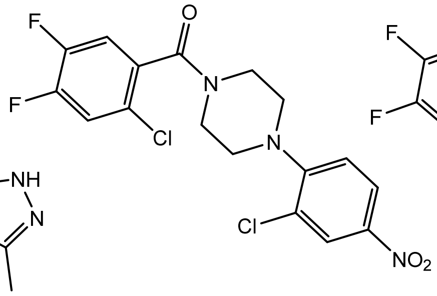
**30**

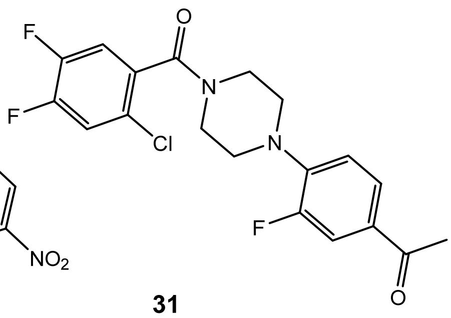
**31**

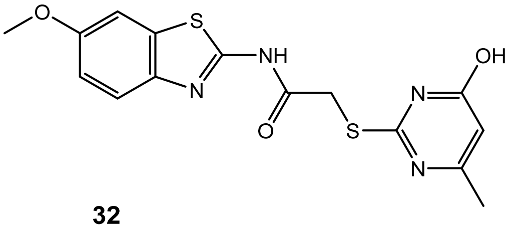
**32**

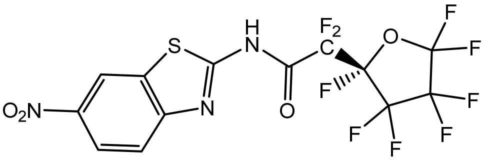
**33**

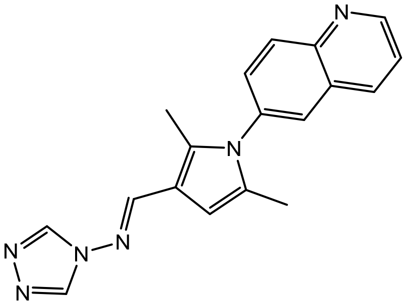
**34**

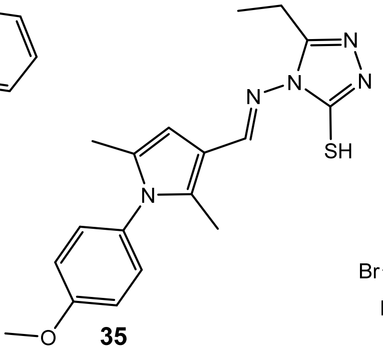
**35**

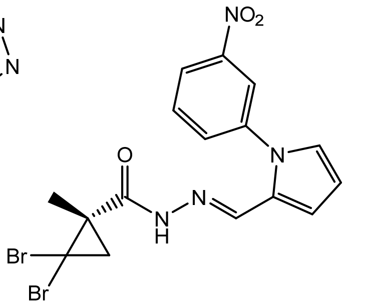
**36**

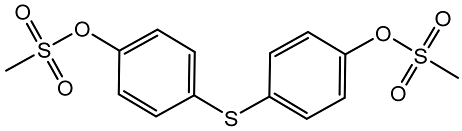
**37**

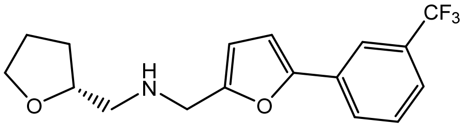
**38**

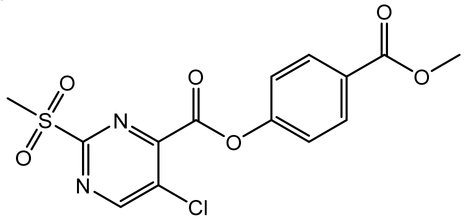
**39**

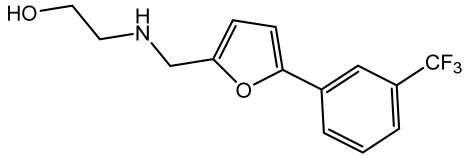
**40**

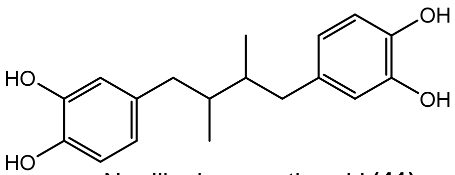
Nordihydroguaretic acid **(41)**

**Supplementary Figure 2.** Chemical structures of selected virtual hits from group 2; nordihydroguaretic acid (**41**) is a secondary plant metabolite, **29**-**40** are synthetic compounds obtained from SPECs.

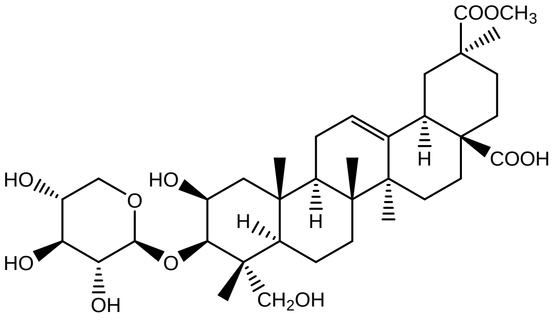

**42**

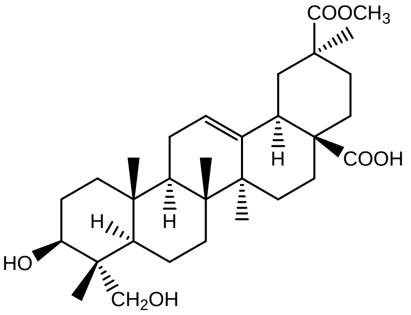

**43**

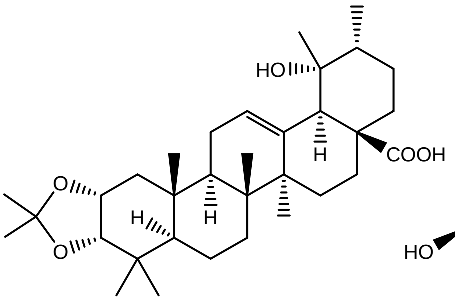

**44**

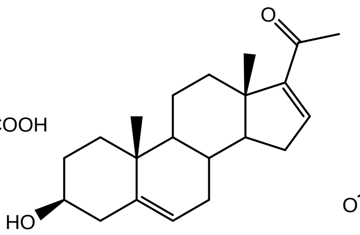

**45**

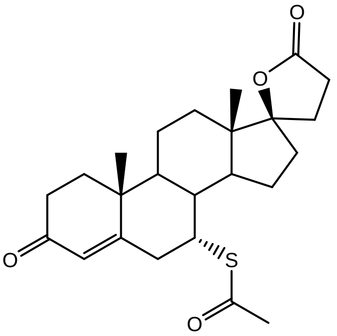

Spironolactone **(46)**

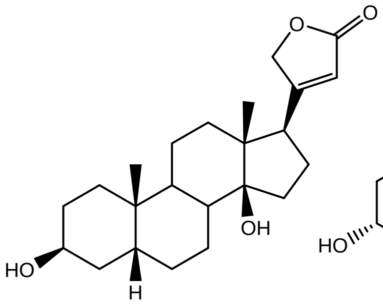

**47**

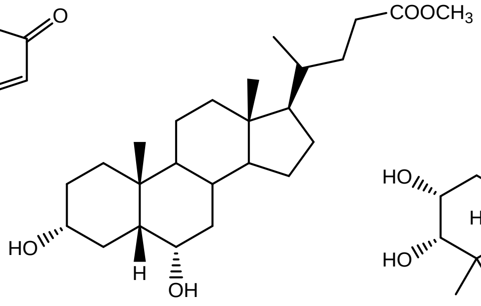

**48**

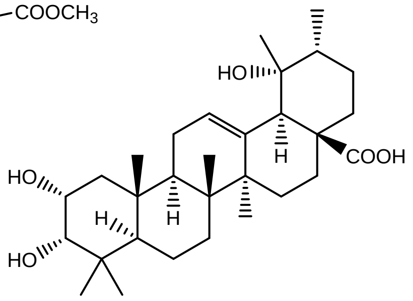

Euscaphic acid **(49)**

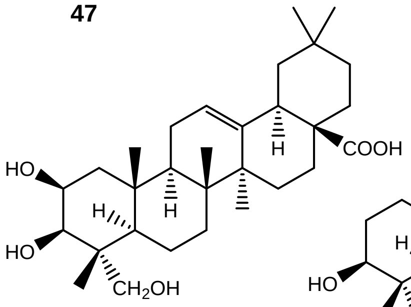

Bayogenin **(50)**

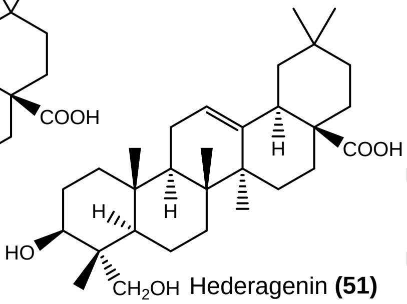

Hederagenin **(51)**

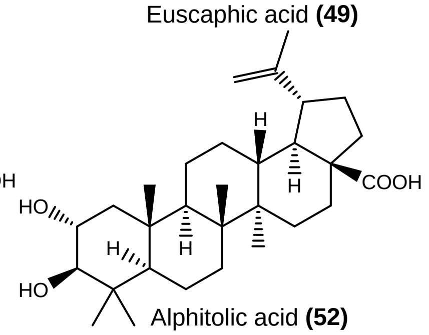

Alphitolic acid **(52)**

**Supplementary Figure 3.** Chemical structures of selected virtual hits from groups 4 to 9. Compounds **42** to **44** and **47** to **52** are secondary plant metabolites, **45** is a progesterone derivative and spironolactone (**46**) is an approved drug.

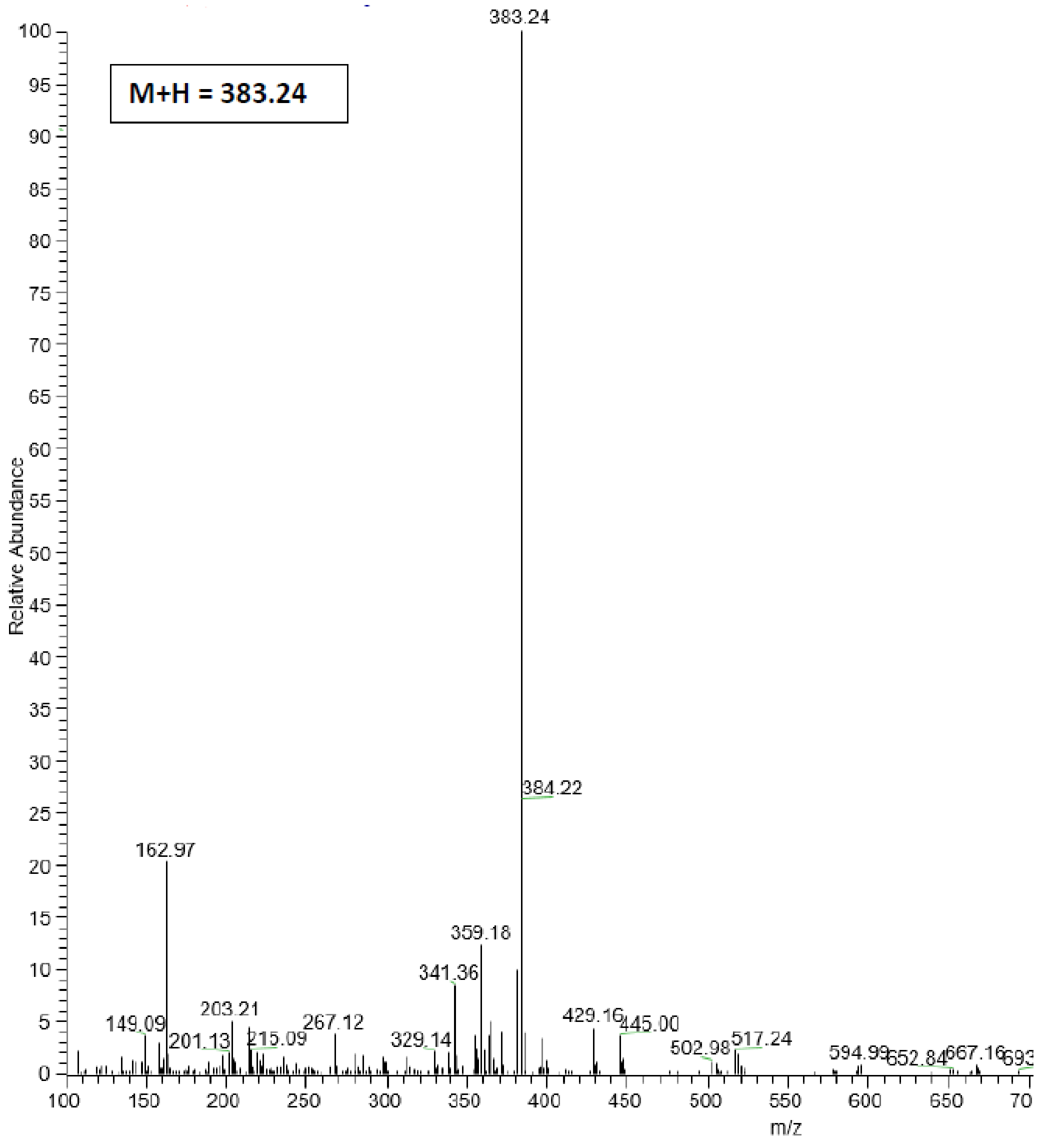


**Supplementary Figure 4.** MS data of microlobidene (**28**) (ThermoLTQiontrap; HESI; 275°C Capillary Temp; 300°C Source Heater Temp; Sheath Gas Flow 40.00; Aux Gas Flow 10.00; Sweep Gas Flow 10.00; Positive Source Voltage 3.70 kV; 100.00 uA Source Current; 18.00 V Capillary Voltage; 90.00 V Tube Lens)

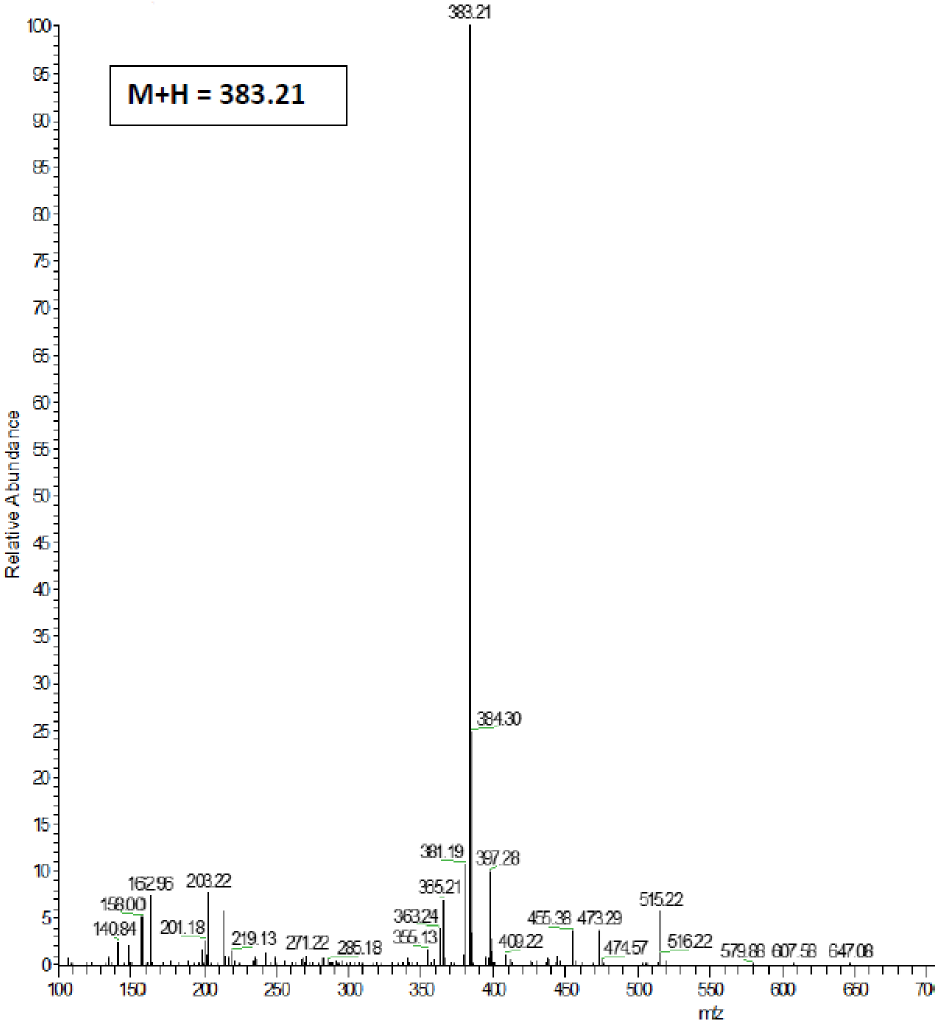


**Supplementary Figure 5.** MS data of farnesiferol B (**27**) (ThermoLTQiontrap; HESI; 275°C Capillary Temp; 300°C Source Heater Temp; Sheath Gas Flow 40.00; Aux Gas Flow 10.00; Sweep Gas Flow 10.00; Positive Source Voltage 3.70 kV; 100.00 uA Source Current; 18.00 V Capillary Voltage; 90.00 V Tube Lens)

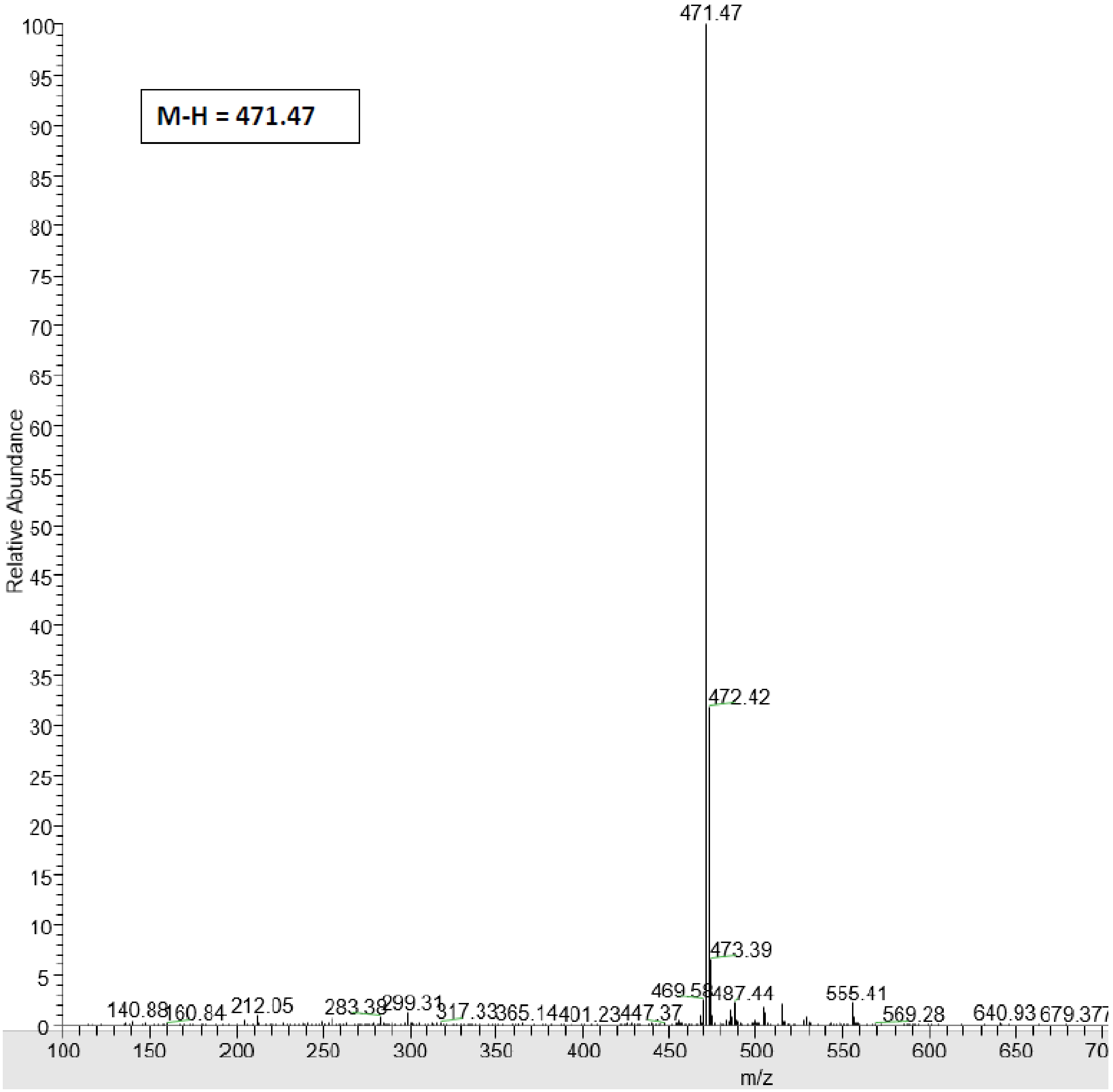


**Supplementary Figure 6.** MS data of alphitolic acid (**52**) (ThermoLTQiontrap; HESI; 275°C Capillary Temp; 300°C Source Heater Temp; Sheath Gas Flow 40.00; Aux Gas Flow 10.00; Sweep Gas Flow 10.00; Positive Source Voltage 3.70 kV; 100.00 uA Source Current; -30.00 V Capillary Voltage; -110.00 V Tube Lens)

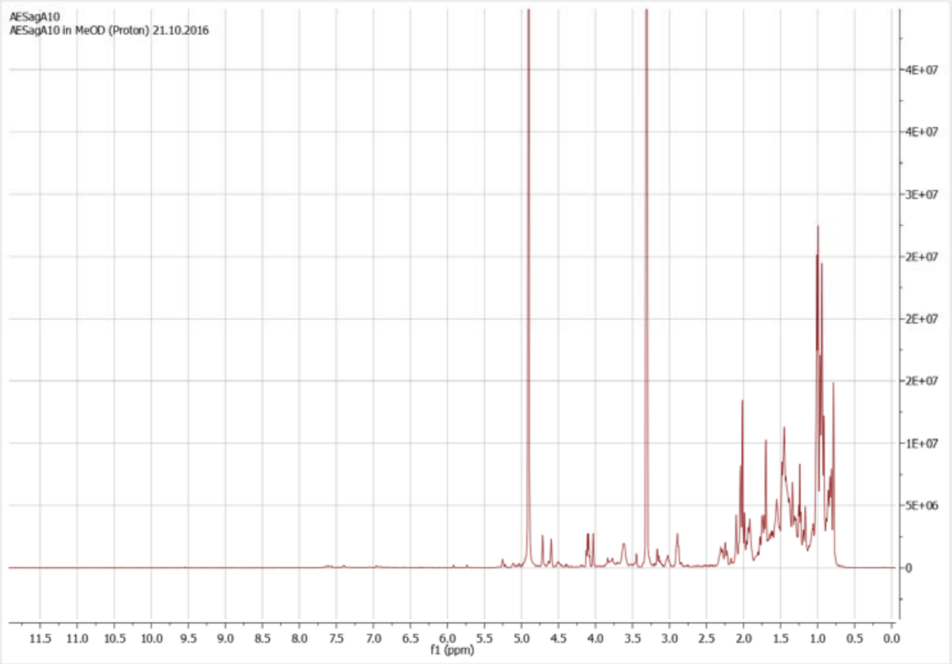


**Supplementary Figure 7.** 1H-NMR spectrum of 52 (CD3OD, ref.: δH 3.31 ppm, 500 MHz NMR, ~2 mg)

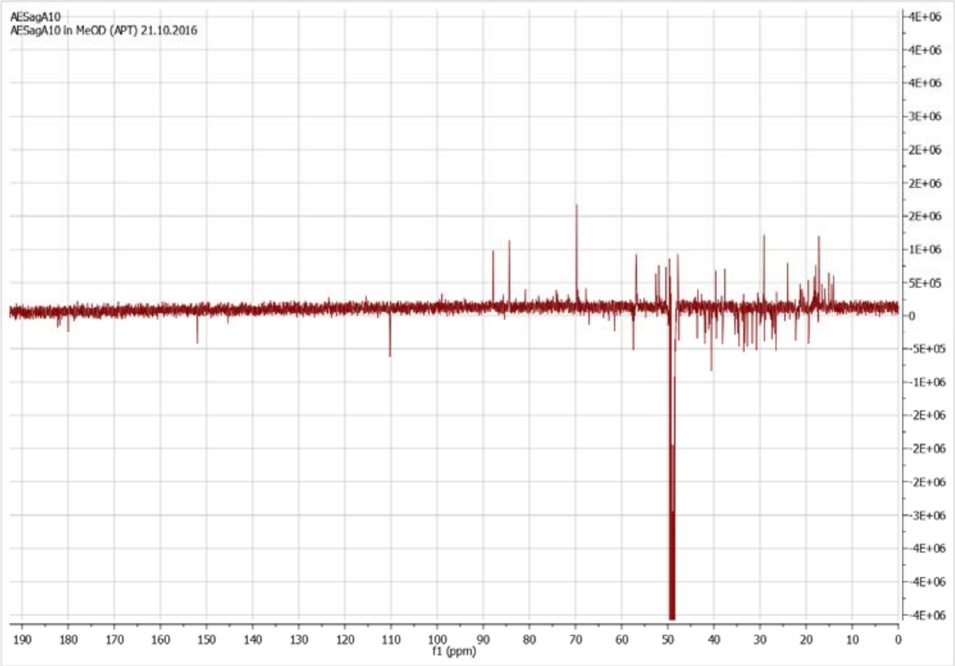


**Supplementary Figure 8.** 13C-APT spectrum of 52 (CD3OD, ref.: δC 49.00 ppm, 500 MHz NMR, ~2 mg)

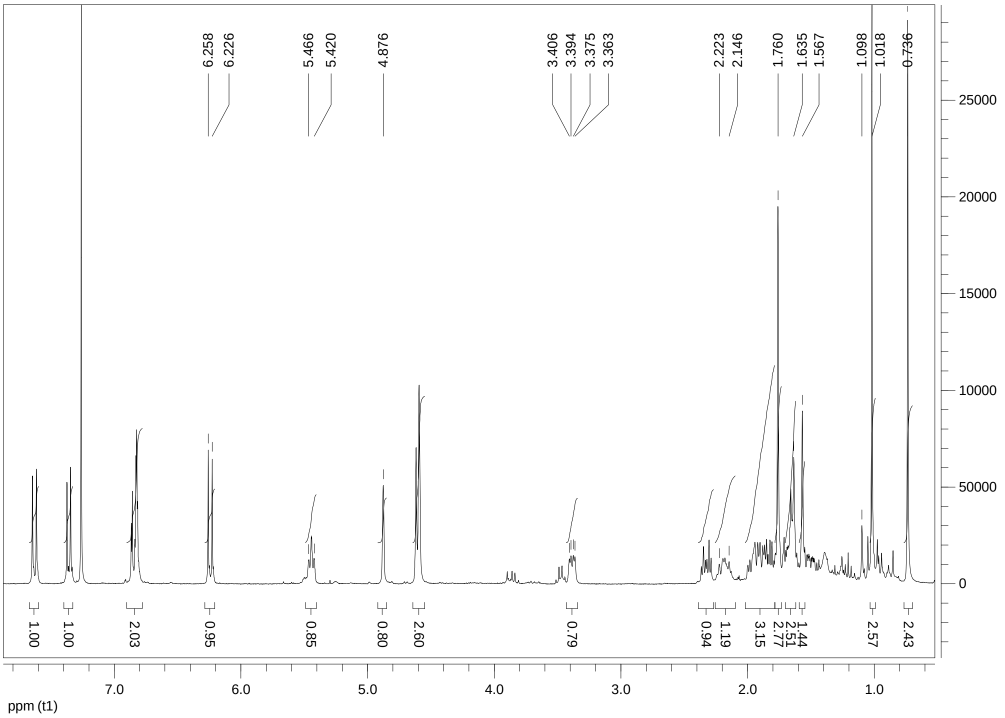


**Supplementary Figure 9.** $^{1}$H-NMR of **27** ($CDCl_3$, ref.: δH 7.26 ppm, 300 MHz NMR, 5.6mg)

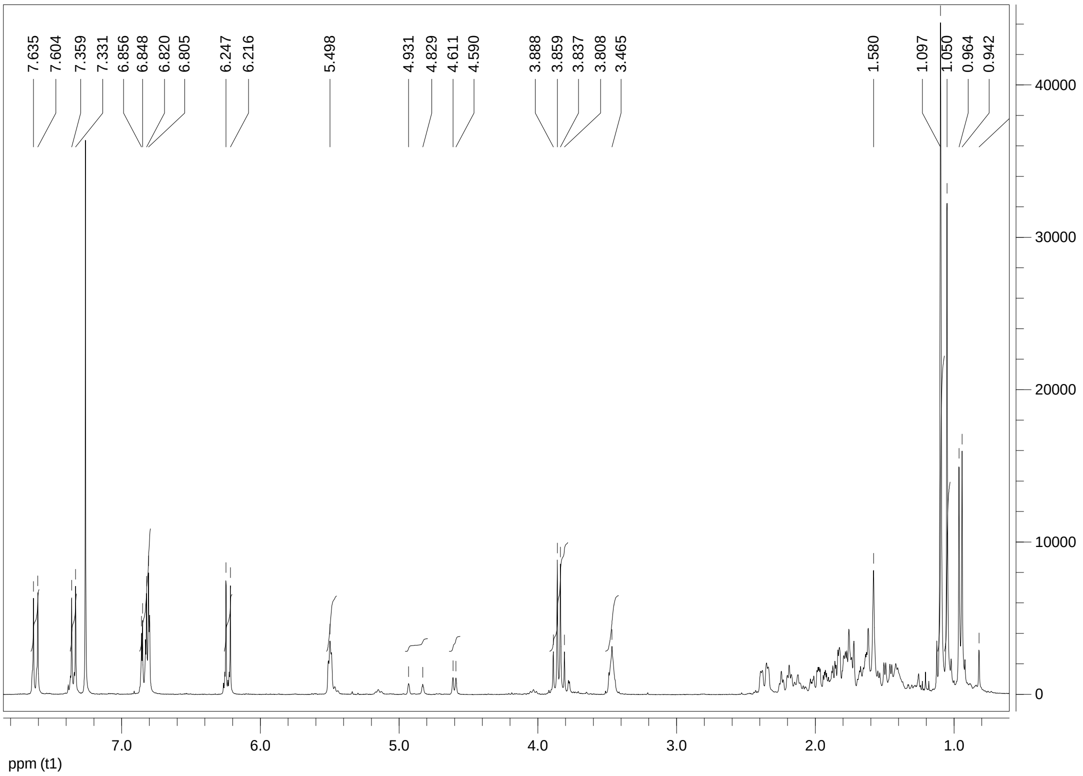


**Supplementary Figure 10.** $^{1}$H-NMR of **28** ($CDCl_3$, ref.: δH 7.26 ppm, 300 MHz NMR, 5.6mg)
